# Effect of early local cold compression therapy combined with rehabilitation exercises on local swelling and joint function recovery in patients with periarticular ankle fractures

**DOI:** 10.12669/pjms.42.9.15793

**Published:** 2026-09

**Authors:** Yanan Zhao, Tiantian Wang, Yulin Liu, Li Zhu, Yuanyuan Liu

**Affiliations:** 1 Yanan Zhao, Baoding Key Laboratory of Mechanisms and Integrated Chinese and Western Medicine Interventions for Orthopedic Perioperative Complications, Baoding 071000, Hebei Province, P.R. China; 2 Tiantian Wang, Baoding Key Laboratory of Mechanisms and Integrated Chinese and Western Medicine Interventions for Orthopedic Perioperative Complications, Baoding 071000, Hebei Province, P.R. China; 3 Yulin Liu, Baoding Key Laboratory of Mechanisms and Integrated Chinese and Western Medicine Interventions for Orthopedic Perioperative Complications, Baoding 071000, Hebei Province, P.R. China; 4 Li Zhu, Baoding Key Laboratory of Mechanisms and Integrated Chinese and Western Medicine Interventions for Orthopedic Perioperative Complications, Baoding 071000, Hebei Province, P.R. China; 5 Yuanyuan Liu, Baoding Key Laboratory of Mechanisms and Integrated Chinese and Western Medicine Interventions for Orthopedic Perioperative Complications, Baoding 071000, Hebei Province, P.R. China

**Keywords:** Early cold compression therapy, Periarticular ankle fracture, Rehabilitation exercise, Swelling, Pain

## Abstract

**Objectives:**

To evaluate the effect of early local cold compression therapy combined with rehabilitation exercises on local swelling and joint function in patients with periarticular ankle fractures.

**Methodology:**

This was a retrospective study. A total of 60 patients with periarticular ankle fractures admitted to Baoding No.1 Central Hospital between June 2023 to June 2024 were enrolled in this study. The participants were divided into an observation group and a control group. The control group received conventional treatment (including bed rest, limb elevation, and intravenous sodium aescinate per institutional protocol) and functional exercises. The observation group received additional early local cold compression therapy (ice-water mixture, 20 minute sessions every four hours for 72 hours post-admission and post-surgery). Ankle swelling, pain intensity, range of motion (ROM), and the American Orthopaedic Foot and Ankle Society (AOFAS) scores were compared between groups.

**Results:**

Swelling and pain significantly decreased in both groups at all postoperative time points vs. baseline, with the observation group showing greater reductions(*P*< 0.05). Dorsiflexion, plantarflexion ROM, and AOFAS scores significantly increased in both groups, with the observation group showing greater improvement (*P* < 0.05).

**Conclusion:**

Early local cold compression therapy combined with rehabilitation exercises can significantly alleviate ankle swelling and pain, improve ankle function as assessed by the AOFAS Ankle-Hindfoot Scale, and accelerate the recovery of ankle ROM in patients with periarticular ankle fractures.

## INTRODUCTION

The ankle joint, primarily consisting of the distal tibia, distal fibula, and the body of the talus, is crucial for walking. Given its inherent instability, the ankle joint is highly susceptible to injuries and even fractures under external forces, often accompanied by ligament damage and joint dislocations.^1^ Ankle fractures account for 7.6% of all fractures and 46.7% of tibiofibular fractures.^2^ Inappropriate treatment can severely impair the functional mobility of the ankle joint. Therefore, the treatment of ankle fractures, whether conservative or surgical, demands precise fracture reduction. Of the entire treatment process, fracture reduction is merely the first step; the restoration of ankle joint function is a longer and more critical process.^3^

Currently, clinical research on ankle fractures mainly focuses on functional recovery. This is because dysfunction of the ankle joint has a strong impact on quality of life.^4^ Early post-fracture movement can lead to ankle swelling and pain. Some patients may show resistance to rehabilitation exercises, which, in turn, affects the recovery of ankle function.^5^ Clinically, the treatment of ankle fractures should fully consider the complexity of the injury, rather than merely achieving anatomical reduction. A holistic approach is required to restore both the structure and function of the ankle joint. Early recovery of ankle joint function allows patients to be discharged sooner, return to normal work and life more quickly, improve their quality of life, and achieve better long-term outcomes. Additionally, clinicians can reduce the occurrence of various complications during treatment. This study primarily investigated the effect of early local cold compression therapy combined with rehabilitation exercises on local swelling and functional recovery in patients with ankle fractures.

## METHODOLOGY

This was a retrospective study. Sixty patients with ankle fractures admitted to Baoding No.1 Central Hospital between June 2023 and June 2024 were selected as the study subjects and divided into two groups based on the intervention method they received: the observation group and the control group, with 30 patients in each group. The two groups were matched for baseline characteristics including age, sex, fracture type (Danis-Weber classification), internal fixation method, and preoperative waiting time. No significant differences were found, ensuring good comparability (Results section, baseline characteristics).

### Ethical approval:

The study was approved by the Institutional Ethics Committee of Baoding No.1 Central Hospital (No.: [2023]011; Approval Date: June 12, 2023), and written informed consent was obtained from all participants.

### Inclusion criteria:

- A confirmed diagnosis of ankle fractures based on X-ray or computed tomography findings.
- Unilateral, first-time ankle fractures.
- Surgical treatment with the incision achieving grade A healing.
- Provision of signed informed consent.

### Exclusion criteria:

- Open fractures of the ankle joint.
- Skin defects or severe soft tissue contusions at the fracture site.
- Presence of severe cardiovascular diseases, liver or kidney dysfunction, malignancies, or other conditions contraindicated for surgery.
- Existing bone and joint system diseases or inflammatory conditions of the ankle joint.
- Pregnant or breastfeeding women, or patients with mental disorders.

### Methods:

The control group received conventional treatment and rehabilitation exercises. Conventional treatment included bed rest and pain management education at admission and after surgery. The affected limb was elevated above the level of the heart using a cushion to reduce swelling. For anti-swelling treatment, according to our institution’s standard protocol during the study period, 10 mg of injectable sodium aescinate was dissolved in 100 mL of 0.9% sodium chloride injection and administered intravenously twice daily. Rehabilitation exercises were as follows: On the day of admission and postoperative day one, local muscle contraction training was initiated. At postoperative day three, patients were instructed to perform active ankle flexion and extension exercises for a total 20 minutes per day, divided into several short sessions (e.g., 5-10 minutes each, 2-4 times per day), with the duration of each session adjusted according to the patient’s pain tolerance, five times daily. At postoperative day four, limited ankle rotation exercises were introduced, with five sessions per day, each lasting 20 minutes. At two weeks post-surgery, patients were instructed to begin non-weight-bearing exercises.

Following their four-week follow-up visit for X-ray review, patients were advised to gradually increase the weight-bearing training at home. The observation group received early cold compression therapy in addition to the conventional treatment provided to the control group. The cold compression therapy was initiated within two hours of admission (preoperatively) and within two hours of the patient returning to the ward postoperatively. An ice-water mixture (ratio of one part ice to two parts water) was placed in a dedicated cryotherapy bag, wrapped in a pure cotton towel, and applied to the ankle joint. Each session lasted 20 minutes and was repeated every four hours for the first 72 hours post-admission and post-surgery. Cold compression therapy was applied immediately after each rehabilitation exercise session. During the cold compression therapy, the skin condition at the treatment site was monitored every 10 minutes. If pallor, cyanosis, or the patient experienced pain or numbness, the ice compress was immediately stopped. The duration and frequency of cold compression therapy could be adjusted as appropriate, and treatment was discontinued for severe reactions.

### Data collection:

Data were retrospectively collected from the hospital’s electronic medical record system. All baseline characteristics (age, sex, injury side, fracture type, fixation method, preoperative waiting time) were extracted by two independent research assistants. Outcome measures (swelling, VAS, ROM, AOFAS) were assessed at predefined time points: before cold compression therapy, preoperatively, and at postoperative day one, week one, and week two. Ankle ROM and AOFAS scores were measured by a trained physiotherapist who was blinded to the group assignment.

### Outcome measures:

- ***Swelling and Pain Levels:*** Swelling and pain levels in the affected ankle joint area were measured at several time points, namely, before cold compression therapy, preoperatively, and at postoperative day one, week one, and week two. The swelling was quantified as the difference in circumference between the affected side (5 cm above the medial malleolus) and the corresponding site on the healthy side. Pain intensity was assessed using the Visual Analogue Scale (VAS), with higher scores indicating more severe pain (0 = no pain and 10 = highest level of pain).
- ***Ankle Joint Range of Motion (ROM):*** Active dorsiflexion and plantarflexion measurements of the affected ankle joint were recorded at postoperative day one, week two, and week four, with patients in a supine position.
- ***Ankle Function:*** Ankle function was assessed at postoperative day one, week two, and week four using the American Orthopaedic Foot and Ankle Society (AOFAS) scoring system, which evaluates five dimensions of ankle function: joint stability, ROM, walking ability, gait, and pain. The total AOFAS score ranges from 0 to 100, with higher scores indicating better functional recovery.

### Statistical analysis:

Data analysis was performed using SPSS 22.0. Normally distributed measurement data were expressed as mean ± standard deviation (*x̅*±*s*), and comparisons were examined using the independent sample t-test. Non-normally distributed data were presented as median (interquartile range) (M [Q1, Q3]), and comparisons were analyzed by the non-parametric Mann-Whitney U test. Categorical data were denoted as counts and frequencies (*n*[%]), and intergroup comparisons were performed using the chi-square (χ²) test. A *P*-value of <0.05 was considered statistically significant. The total follow-up period for the primary outcome measures in this study was two weeks postoperatively. All patients returned for an X-ray review at four weeks post-surgery to guide the gradual increase of weight-bearing training.

## RESULTS

The control group included 20 males and 10 females, mean age 52.37 ± 15.72 years. The observation group included 19 males and 11 females, mean age 54.90 ± 15.12 years. No significant differences in baseline characteristics (age, sex, injured side, fracture type, fixation method) were observed between the two groups (all P > 0.05). Furthermore, the preoperative waiting time (days from admission to surgery) was comparable between the control group (4.2 ± 1.3 days) and the observation group (4.1 ± 1.5 days; P = 0.781), indicating good baseline comparability.

Before cold compression therapy, there were no significant differences in swelling and pain levels between the two groups (both *P >* 0.05). After cold compression therapy, at preoperative, postoperative day one, week one, and week two, both groups showed significant reductions in swelling and pain levels compared with baseline. Furthermore, the observation group demonstrated a significantly greater reduction than the control group (*P <* 0.05). Table-I, Fig.1 and 2.

**Table-I:** Comparison of swelling and pain levels between the two groups at different time points (*x̅*±*s*).

Parameter	Before cold compression therapy	Preoperative	Postoperative day one	Postoperative week one	Postoperative week two
***Swelling level (cm)***					
Control (n = 30)	3.79±0.74	2.89±0.56	3.29±0.57	2.70±0.59	1.68±0.61
Observation (n = 30)	3.72±0.79	2.54±0.51	2.86±0.52	2.06±0.53	1.14±0.54
*t-value*	0.388	2.507	3.016	4.449	3.647
*P-value*	0.700	0.015	0.004	<0.001	0.001
***VAS score***					
Control (n = 30)	7.97±0.72	5.87±0.82	7.50±0.51	3.00±0.91	1.17±0.79
Observation (n = 30)	7.90±0.66	5.13±0.78	7.03±0.67	2.47±0.63	0.77±0.57
*t-value*	0.374	3.559	3.043	2.641	2.248
*P-value*	0.710	0.001	0.004	0.011	0.028

**Fig.1:**
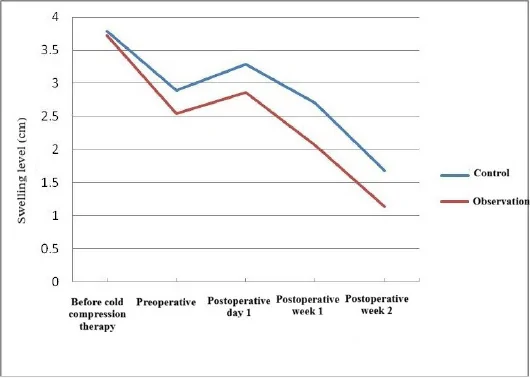
Comparison of swelling levels between the two groups at different time points.

**Fig.2:**
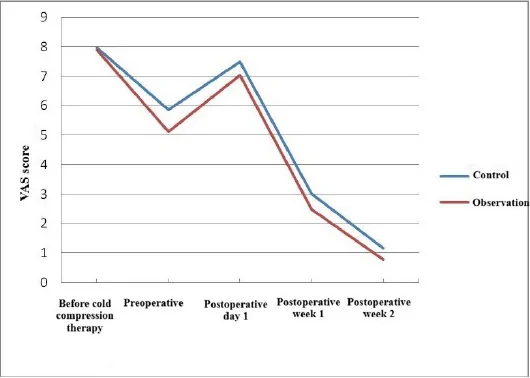
Comparison of VAS scores between the two groups at different time points.

Before cold compression therapy, there were no significant differences in dorsiflexion and plantarflexion angles between the two groups (both *P >* 0.05). After cold compression therapy, at preoperative, postoperative day one, week one, and week two, both groups showed significant increases in dorsiflexion and plantarflexion ROM compared with baseline. Moreover, the observation group demonstrated a more pronounced improvement than the control group (*P <* 0.05). Table-II, Fig.3 and 4.

**Table-II:** Comparison of ankle joint ROM between the two groups at different time points (*x̅*±*s*).

Parameter	Before cold compression therapy	Preoperative	Postoperative day one	Postoperative week one	Postoperative week two
***Dorsiflexion angle (°)***					
Control (n = 30)	11.30±1.49	14.87±1.83	9.73±1.72	15.47±1.53	18.33±1.42
Observation (n = 30)	11.53±1.36	15.93±1.55	10.67±1.40	16.53±1.36	19.67±1.21
*t-value*	0.634	2.432	2.306	2.861	3.907
*P-value*	0.528	0.018	0.025	0.006	<0.001
***Plantarflexion angle (°)***					
Control (n = 30)	22.17±1.15	32.73±2.53	24.23±1.22	34.90±1.18	38.73±1.31
Observation (n = 30)	21.97±1.61	34.83±2.31	25.97±2.28	35.60±1.16	40.37±1.50
*t-value*	0.555	3.359	3.667	2.310	4.496
*P-value*	0.581	0.001	0.001	0.024	<0.001

**Fig.3:**
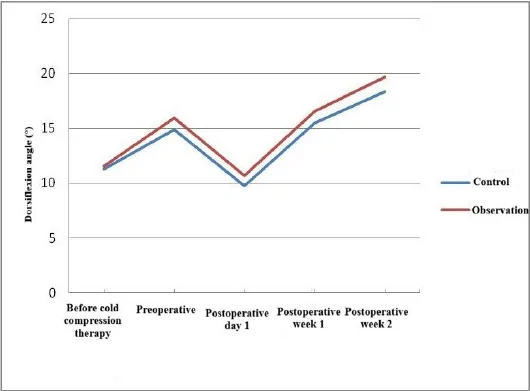
Comparison of dorsiflexion ROM between the two groups at different time points.

**Fig.4:**
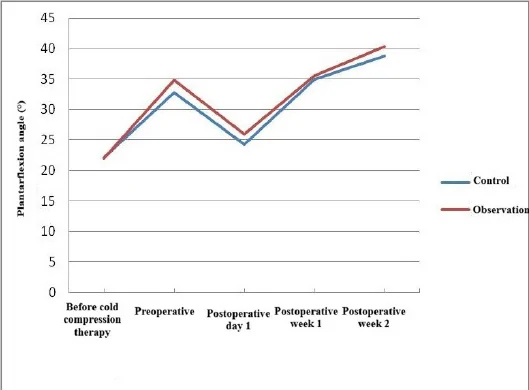
Comparison of plantarflexion ROM between the two groups at different time points.

Before cold compression therapy, there were no significant differences in the AOFAS score between the two groups (*P >* 0.05). After cold compression therapy, at preoperative, postoperative day one, week one, and week two, both groups demonstrated significant increases in the AOFAS score compared with baseline. The observation group showed a significantly greater improvement than the control group (*P <* 0.05). Table-III and Fig.5.

**Table-III:** Comparison of AOFAS scores between the two groups at different time points (*x̅*±*s*).

Group	Before cold compression therapy	Preoperative	Postoperative day one	Postoperative week one	Postoperative week two
Control (n = 30)	51.93±0.83	65.43±1.17	60.37±1.10	78.33±2.31	84.90±1.30
Observation (n = 30)	52.17±0.65	74.80±2.64	66.63±2.55	80.23±1.52	88.47±1.50
*t-value*	1.216	17.754	12.352	3.761	9.846
*P-value*	0.229	<0.001	<0.001	<0.001	<0.001

**Fig.5:**
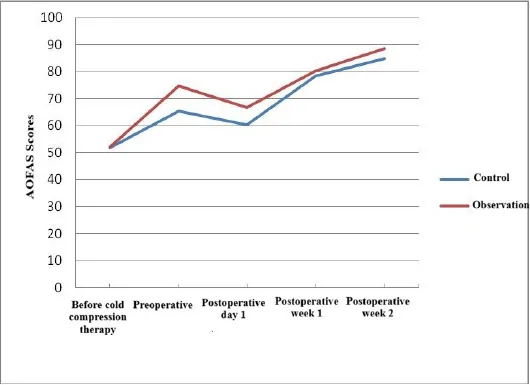
Comparison of AOFAS scores between the two groups at different time points.

## DISCUSSION

This study demonstrated that early local cold compression therapy combined with rehabilitation exercises significantly alleviated ankle swelling and pain, improved ankle function (AOFAS score), and accelerated the recovery of ankle ROM in patients with periarticular ankle fractures. Specifically, our key findings include:

- the observation group showed a significantly greater reduction in ankle circumference and VAS scores at all postoperative time points compared to the control group;
- dorsiflexion and plantarflexion ROM recovered more rapidly in the observation group; and (3) the AOFAS scores, a comprehensive measure of ankle function, were significantly higher in the observation group starting from the preoperative assessment.

These findings are consistent with several previous studies.^6^-^10^ Reported similar benefits of cryotherapy in reducing postoperative edema and pain after ankle fracture surgery.^6^,^11^ A systematic review also confirmed that postoperative cryotherapy is effective in reducing pain and analgesic use in ankle surgery.^12^ Our results align with these reports, providing further evidence for the clinical utility of cold compression.^13^ However, our study goes beyond by specifically integrating cold compression with a structured, early rehabilitation exercise protocol, demonstrating that the combination is feasible and effective in a clinical setting. Our findings are also in agreement with a study who used infrared irradiation and kinesiology tape for similar purposes,^14^ but our cold compression method is simpler, more cost-effective, and can be easily administered by patients or their families.

The mechanism by which cold compression therapy improves outcomes is likely multifactorial.^15^ We propose that the cooling effect induces local vasoconstriction, reducing microvascular permeability and limiting the formation of edema.^16^ This reduction in swelling, in turn, alleviates pain by decreasing pressure on local nerve endings.^17^ Furthermore, by mitigating the post-traumatic and postoperative inflammatory response, cold compression may limit secondary tissue damage, thereby facilitating earlier and less painful participation in rehabilitation exercises. This is crucial because early mobilization is known to prevent joint stiffness, muscle atrophy, and adhesions.^18^ Notably, our results show that the AOFAS score, despite a temporary decrease at postoperative day one, remained higher than pre-treatment levels in the observation group. This suggests that the preemptive application of cold compression therapy before surgery might prime the joint for a better recovery trajectory.

### Strengths of our study:

It include its comprehensive assessment of outcomes, combining objective measures (swelling, ROM) with validated patient-reported scales (VAS, AOFAS). The clear description of our cold compression protocol (ice-water mixture, specific timing and frequency) allows for easy replication. Moreover, we demonstrate that patient compliance with rehabilitation exercises was subjectively higher in the observation group, likely due to better pain control, which is a crucial but often overlooked factor in functional recovery.^19^

### Limitations:

First, the sample size was relatively small (60 patients) due to the single-center, retrospective nature of the study, which may limit the generalizability of our findings. Therefore, our results should be interpreted as preliminary. Second, only swelling, pain, and ankle ROM were assessed, lacking a broader range of objective measures such as inflammatory cytokines. Third, the postoperative follow-up was relatively short (only two weeks for the primary outcomes), making it impossible to measure long-term outcomes like the incidence of post-traumatic arthritis. Fourth, as a retrospective study, the lack of blinding of patients and treating physicians is a potential source of bias, although the outcome assessors for ROM and AOFAS scores were blinded to group assignment. Fifth, the use of injectable sodium aescinate as a conventional treatment may not represent current clinical practice in all centers, which could limit the direct comparability of our results with studies using updated protocols.

## CONCLUSIONS

Early local cold compression therapy combined with rehabilitation exercises can significantly alleviate swelling and pain in patients with ankle fractures, facilitate early functional rehabilitation training, and accelerate the recovery of ankle joint function.

### Recommendations:

Future large-scale, multi-center randomized controlled trials with longer follow-up periods are needed to confirm these findings and establish optimal protocols.
